# Clinical Decision Support Systems for Brain Tumour Diagnosis and Prognosis: A Systematic Review

**DOI:** 10.3390/cancers15133523

**Published:** 2023-07-06

**Authors:** Teesta Mukherjee, Omid Pournik, Sarah N. Lim Choi Keung, Theodoros N. Arvanitis

**Affiliations:** Department of Electronic, Electrical and Systems Engineering, School of Engineering, College of Engineering and Physical Sciences, University of Birmingham, Edgbaston, Birmingham B15 2TT, UK

**Keywords:** clinical decision support system, brain tumour, brain neoplasms, diagnosis, prognosis, systematic review

## Abstract

**Simple Summary:**

Brain tumours are abnormal growth of cells in the human brain. Continuous effort is being made towards improving diagnosis and treatment options for such brain neoplasms. Manual classification and segmentation of imaging scans are tedious, time-consuming, and subjective. Over the last decade, the use of intelligent systems in the form of clinical decision support systems (CDSSs) to assist in identifying, classifying, and evaluating brain tumours has seen a rise. A CDSS can be used as a supportive tool for clinicians to deal with complex medical decisions and improve healthcare delivery. This review aims to systematically identify different types of CDSSs used in brain tumour diagnosis and prognosis through medical imaging. It analyses various CDSS tool types, techniques, accuracy, and outcomes to provide the latest evidence available in this field of research.

**Abstract:**

CDSSs are being continuously developed and integrated into routine clinical practice as they assist clinicians and radiologists in dealing with an enormous amount of medical data, reduce clinical errors, and improve diagnostic capabilities. They assist detection, classification, and grading of brain tumours as well as alert physicians of treatment change plans. The aim of this systematic review is to identify various CDSSs that are used in brain tumour diagnosis and prognosis and rely on data captured by any imaging modality. Based on the 2020 preferred reporting items for systematic reviews and meta-analyses (PRISMA) protocol, the literature search was conducted in PubMed and Engineering Village Compendex databases. Different types of CDSSs identified through this review include Curiam BT, FASMA, MIROR, HealthAgents, and INTERPRET, among others. This review also examines various CDSS tool types, system features, techniques, accuracy, and outcomes, to provide the latest evidence available in the field of neuro-oncology. An overview of such CDSSs used to support clinical decision-making in the management and treatment of brain tumours, along with their benefits, challenges, and future perspectives has been provided. Although a CDSS improves diagnostic capabilities and healthcare delivery, there is lack of specific evidence to support these claims. The absence of empirical data slows down both user acceptance and evaluation of the actual impact of CDSS on brain tumour management. Instead of emphasizing the advantages of implementing CDSS, it is important to address its potential drawbacks and ethical implications. By doing so, it can promote the responsible use of CDSS and facilitate its faster adoption in clinical settings.

## 1. Introduction

Brain tumours are abnormal and uncontrolled growth of cells in the human brain that affect usual brain functionality [1]. Brain tumours are divided into primary and secondary. Primary brain tumours originate in the brain and can be subdivided into benign (non-cancerous) and malignant (cancerous). Secondary brain tumours are cancerous cells expanding to the brain from other parts of the human body [2]. The World Health Organization (WHO) classifies brain tumours into four grades. Grades 1 and 2 consist of less severe tumours such as meningiomas, while Grades 3 and 4 consist of more severe ones such as gliomas [3]. Management and treatment of these brain neoplasms require an understanding of the location, size, and type of tumour. Various imaging modalities such as magnetic resonance imaging (MRI), positron emission tomography (PET), and computed tomography (CT) are used in the diagnosis of brain tumours. MRI is usually preferred as it is non-ionizing and non-invasive [4]. However, manual segmentation and classification of these images are tedious processes, are prone to human error, and can be subjective. To address these challenges, clinical decision support systems (CDSSs) are being used as a supportive tool for radiologists and clinicians to aid in the diagnosis and prognosis of brain tumours [4].

**Figure 1:**
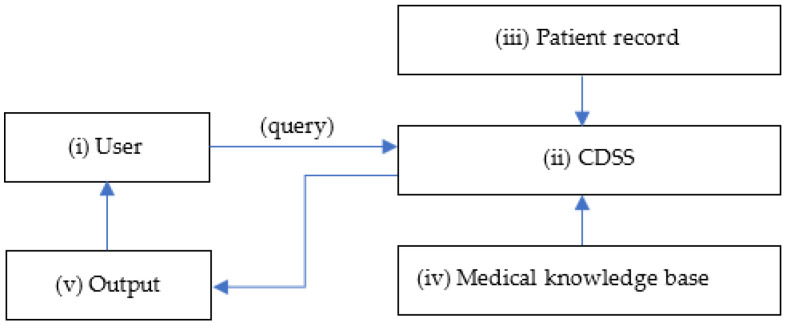
Simple diagram of a CDSS. (**i**) User at the point of care sends a healthcare query to the (**ii**) CDSS that matches (**iii**) patient record with the (**iv**) medical knowledge base and responds with an (**v**) output with clinical recommendations.

CDSSs are primarily designed for clinicians to use at the point of care, as shown in Figure 1. A conventional CDSS is comprised of software designed to match patient characteristics with a computerised medical knowledge base and present a patient-specific recommendation or evaluation to the clinician for making an informed decision [5]. These computerised systems aid in early detection and characterisation of brain tumours by performing automatic tumour segmentation, differentiation, classification, and evaluation of brain imaging data [6].

Available literature on CDSSs, used specifically for brain tumours, is limited. This systematic review is, to the best of our knowledge, the first of its kind to evaluate different types of CDSSs used both for brain tumour diagnosis and prognosis through medical imaging data. The research question is to identify what CDSSs are being used in the diagnosis and prognosis of brain tumours, to summarise the techniques used, and to evaluate their accuracy and outcomes.

## 2. Method

The methodology has been divided into (i) search strategy—databases used; (ii) study selection—keywords and inclusion and exclusion criteria; (iii) data extraction—pre-defined data extraction proforma; (iv) study quality assessment—to assess the quality of included studies; (v) data synthesis—reasons for conducting a narrative and semi-quantitate review.

### 2.1. Search Strategy

The literature search was conducted, systematically, in two academic databases, viz., PubMed and Engineering Village. Both databases provided all the relevant studies needed in this area of research. For identifying medical literature in PubMed, medical subject headings (MeSH) terms were used—(“decision support systems, clinical” [MeSH Terms] AND “Brain Neoplasms” [MeSH Terms]). In Engineering Village, both controlled vocabulary and general terms were used—(((((((cancer)) OR ((((({Tumors} WN CV) OR ({Oncology} WN CV))))))) AND ((((({Decision support systems} WN CV))))))) AND brain). Studies published only in the English language were considered in this review. The search was not bound by any time frame.

### 2.2. Study Selection

**Figure 2:**
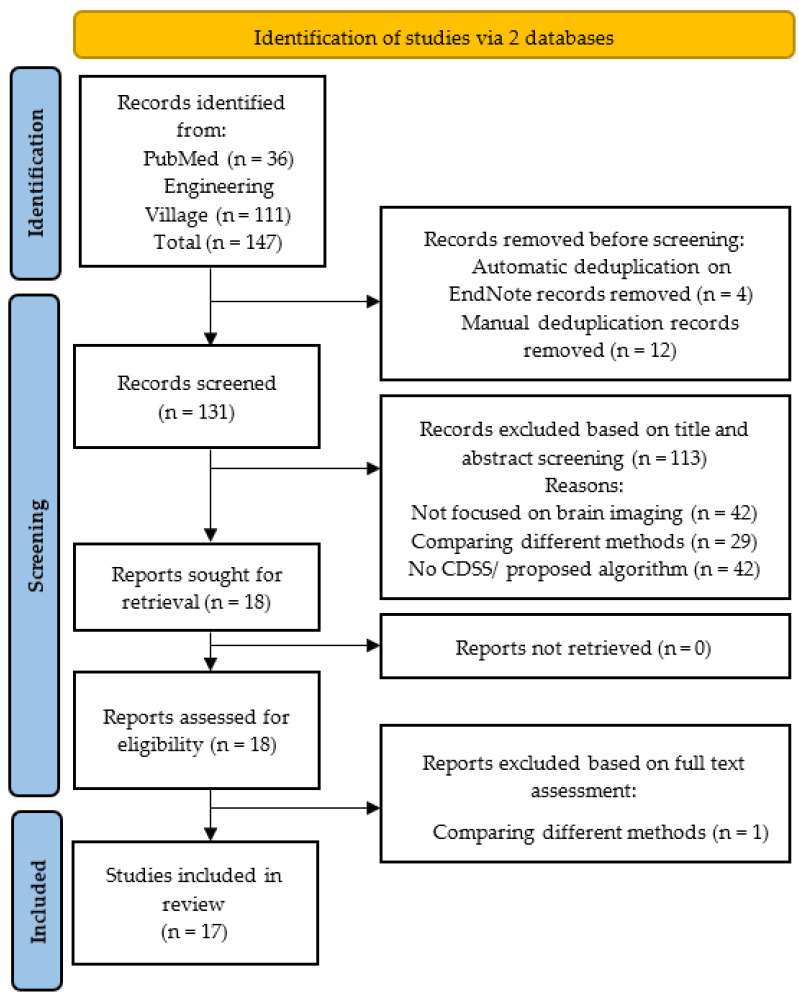
PRISMA flow diagram for study selection.

The systematic review was conducted according to the 2020 PRISMA protocol [7]. The PRISMA flowchart is shown in Figure 2. The literature search was conducted without any time frame to identify all studies published until May 2023.

Based on the purpose of this systematic review, there were 4 exclusion criteria: (i) studies that did not use CDSS; (ii) studies that focused on comparing different methods/techniques—comparative studies are out of scope of the current review; (iii) studies that did not investigate brain tumours; and (iv) studies that focused on treatment options for brain tumours, such as surgery or radiotherapy, as opposed to focusing on the use of CDSSs in the management of tumours. To provide an unbiased and comprehensive summary of CDSSs used in the diagnosis and prognosis of brain tumours, papers that had insufficient information on results or limited/poor methodology were also excluded. These comprised studies with inadequate sample sizes, flawed study designs, or biased data collection methods.

### 2.3. Data Extraction

Two authors with in-depth knowledge of digital health technology and experience in conducting systematic reviews independently performed data extraction using a pre-defined data extraction proforma. Any conflicts or discrepancies between these two authors were resolved by a third reviewer who is a senior researcher and an acknowledged expert in the field of digital healthcare and neuro-oncology imaging clinical decision support systems. Rayyan, a web application designed to facilitate the screening process in systematic reviews, was utilised to track and manage conflicts. Discrepancies arose in a small fraction of cases (6%), which were resolved through a consensus. Variables used for extraction of data were the year of study publication, study design, geographical location of the research conducted, sample size, modality used, CDSS features, techniques/methods used, and CDSS output. These have been listed in Table 1 and Table 2 below.

### 2.4. Study Quality Assessment

All studies included in this review have been assessed for the quality of their research. Keshav’s 5 Cs (viz., category, context, clarity, correctness, and contribution) [8] framework was used to ensure a comprehensive approach towards including papers in this review. Additionally, only studies that had adequate reasons for determining their sample size, patient selection criteria, and methodology were considered. This reduces bias and improves the overall quality of a systematic review.

### 2.5. Data Synthesis

The identified studies were diverse in terms of their sample size, type of CDSS, and techniques used. Hence, a meta-analysis was not performed. Rather, both a narrative and semiquantitative summary of CDSSs used in brain tumour diagnosis and prognosis has been provided. Individual CDSSs have been broadly categorised, as and when necessary.

**Table 2:** Type of CDSS, modalities, techniques, accuracy, and outcome.

Ref	CDSS Description	Sample Size	Modality	Brain Tumour Types	Techniques Used	Accuracy	Outcome
[9]	Data-driven prognostic support	42	Fluid-attenuated inversion recovery (FLAIR) or T2-weighted MRI	Diffuse low-grade gliomas	Linear and exponential mathematical models with coefficient of determination R^2^ and *t*-test to evaluate quality of model predictions	89.00%	Notifies clinicians of changes in tumour diameter and whether to continue/stop treatment
[10]	Diagnostic support for the detection and classification of tumours	Benign: training 75, testing 65 Malignant: training 75, testing 65	MRI	All	Denoising by the genetic median filter, segmentation by hierarchical fuzzy clustering, feature extraction by GLCM and Gabor feature, feature selection by lion optimization, and classifier by BSVM	97.69%	Analyses size and type of tumour, stage of cancer
[11]	Diagnostic support that identifies and grades tumours in terms of their severity	Hospital: 134, dataset: 80	T1-weighted, T2-weighted, T1 post-contrast and FLAIR MRI	Low-grade and high-grade gliomas	MRI pulse fusion, segmentation by adaptive thresholding, feature extraction by run length matrix, identification and classification by NB classifier	96.47%	Detects and specifies tumours
[12]	Diagnostic support is not integrated but ready to be used at local and remote level	30	3D T1-weighted MRI	All	Segmentation by semi-automated 3D segmentation method, feature extraction by BoW, classification by SVM	99.00%	Provides tumour detection, segmentation and 3D visualisation
[13]	Diagnostic support for detection and classification of tumours	48	T1 post-contrast MRI	Glioblastoma and metastases	Feature extraction by Student’s *t*-test and correlation analysis; classifiers used QDA, NB, k-NN, SVM and NNW	97.92%	Automatically differentiates between glioblastoma multiforme and solitary metastasis
[14]	A multi-stage classifier for MR spectra of brain tumours developed as part of a DSS	81 astrocytoma, 32 metastases, 37 meningioma, 6 oligodendroglioma, 6 lymphoma, 5 primitive neuroectodermal tumour, 4 schwannoma, 4 haemangioblastomas and 14 healthy	^1^H MRS	All	3 diagnostic classifiers used: LDA, decision trees, and k-NN	99.30%	Provides accurate predictions and reduces classification errors
[15]	Diagnostic support for the detection and classification of tumours	-	^1^H MRS	All	Pattern recognition and data visualisation by LDA	90.00%	Non-invasive tumour diagnosis and grading
[16]	Diagnostic support and qualitative evaluation of Curiam BT	55	^1^H MRS	All	Fisher LDA and Peak Integration	>83.00%	Classification and grading of brain tumours
[17]	Diagnostic support: FASMA for brain tumour classification	126	T2-weighted, T1 post-contrast MRI/^1^H MRS, DWI, DTI, PWI	Gliomas, solitary metastases, atypical meningiomas	SVM, LDA, k-NN and NB	>80.00%	Used advanced MRI techniques for brain tumour classification
[18]	Childhood cancer diagnosis by MIROR	48	T1-weighted, T2-weighted MRI/^1^H MRS, DWI	All	SVM and k-NN	89% and 93%	Performs non-region-specific quantitative analysis of brain imaging data
[19]	Diagnostic support for paediatric brain tumour characterisation (part of HealthAgents)	33	^1^H MRS	Pilocytic astrocytoma, ependymoma, medulloblastoma	Principal component analysis, linear discriminant analysis on MRS data	94.00%	Categorises children’s brain tumours
[20]	Diagnostic support for brain tumour diagnosis and prognosis (part of HealthAgents)	182	MRS, ex vivo high-resolution magic angle spinning (HR-MAS)	All	LDA, SVM and LSVM	>90.00%	Diagnosis and management brain tumours
[21]	Diagnostic support automatic classification framework as a part of HealthAgents	-	MRS	All	Classifiers: LDA, KNN, LS-SVM	>80.00%	Classification of brain tumours
[22]	INTERPRET	-	T1 post-contrast, MRS	All	short, long and concatenated short + long TE	89.00%	Diagnosis and grading of tumours
[23]	Diagnostic support and evaluation of INTERPRET 2.0	38	T1 Spin Echo (SE), axial T2 SE, axial FLAIR, axial T1 SE, axial T1 post-contrast, coronal T1-post-contrast and DWI	All	short, long and concatenated short + long TE	87.00%	Classification of brain tumours
[24]	Diagnostic support and evaluation of INTERPRET DSS v3	From INTERPRET: 266 From IDI-Bellvitge: 70	T1-weighted, T2-weighted, ^1^H MRS	All	LDA-based classifiers: short, long and concatenated short + long TE	>69.84%	Categorisation of MRS from abnormal brain mass
[25]	Diagnostic support for the detection and classification of tumours developed by INTERPRET project	334	Axial T2-weighted, axial T1-weighted pre-contrast, axial T1-weighted post-contrast MRI, ^1^H MRS	All	LDA-classifier	>90.00%	Prediction of tumour classes and grading of tumours

## 3. Results

All studies, included in this review, allow us to answer these research questions: What are the available CDSSs for the diagnosis and prognosis of all types of brain tumours, what their features and techniques are, and finally, what their accuracy and outcomes are. These are covered in the following sections.

### 3.1. Search Results

The literature search conducted via two databases produced 146 studies out of which PubMed identified 36, while Engineering Village identified 111 studies. Automatic de-duplication in EndNote removed 4 studies, while a manual scan removed an additional 14 duplicates, leaving 131 studies to be evaluated for the title and abstract screening in Figure 2. Based on the title and abstract screening, 113 articles were removed. Out of the remaining 18 studies for full-text assessment, only 1 did not fulfil all the inclusion criteria and, thus, was removed. Finally, 17 articles were shortlisted for this systematic review.

### 3.2. Study Characteristics

All 17 studies identified were full-text articles (100%); there were no abstracts from conference presentations. The types of study design within the review have been documented in Table 1.

**Figure 3:**
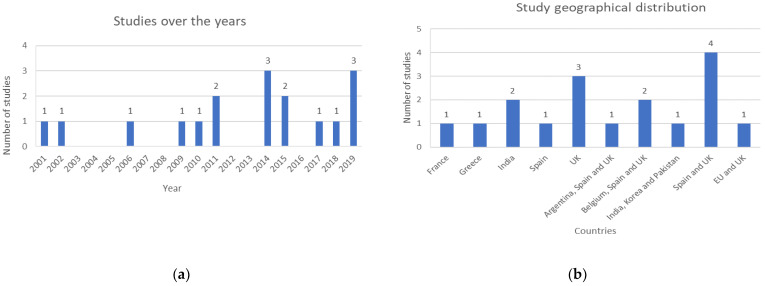
Demographics of included studies: (**a**) Distribution of studies over the years; (**b**) Distribution of study geographies.

The search was not filtered by any time frame, in order to include all available studies in this area until 30 May 2023. The distribution of studies published over time, and their geographical locations are described in Figure 3a and Figure 3b, respectively.

In total, 7 studies (41%) were based on publicly available datasets, 3 studies (18%) were conducted based on international datasets, 2 studies (12%) used regional data, and 5 studies (29%) were conducted at a single centre.

### 3.3. CDSSs Used in the Diagnosis and Prognosis of Brain Tumours

The different types of CDSSs identified through this review, along with the sample size, modality, sub-specific type of brain tumour, techniques, accuracy, and outcome have been listed in Table 2.

#### 3.3.1. Diagnostic Support Systems

MRI-based brain tumour classifier systems were proposed by [10,11]. Both studies utilised the publicly available benchmark Brain Tumor Segmentation (BraTS 2015) dataset, widely used for research on the challenge of brain tumour segmentation. It includes multiple modalities such as T1-weighted, T2-weighted, T1-weighted post contrast, and FLAIR MRI sequences. Features were extracted by using the gray level co-occurrence matrix (GLCM) method and run length of centralized patterns (RLCP), respectively. The accuracy of classification of tumours performed by the boosting support vector machine (BSVM) algorithm was 97.69% [10] as compared to 96.47% using naïve Bayes (NB) [11]. When comparing the two classifiers, BSVM can be considered to have superior capabilities as it performs well even with larger, high-dimensional datasets and the algorithm’s complexity does not increase with reduced training time. Another study by [12], based on a hospital dataset of 30 patients, with an accuracy of 99.00%, was able to determine the size, shape, and location of tumours by utilising the speeded up robust features (SURF) enhanced bag-of-words (BoW) feature extraction method combined with a SVM classifier. The 3D visualisation capability of this CDSS outperformed available state-of-the-art tools, such as ITK-SNAP and 3D-Doctor, according to a subjective comparative analysis. Based on a subjective evaluation undertaken by two separate expert raters, the proposed diagnostic support system can be implemented at local and remote levels. Finally, a study by [13] proposed a computerised decision support framework, with a sample size of 48 patients, for automatic tumour discrimination between glioblastoma multiforme (GBM) and solitary metastasis (MET) using MRI. The novel segmentation method (D-SEG), along with a neural networks-based classifier, achieved an accuracy of 97.92%. However, using a semi-automatic segmentation method and a relatively smaller dataset can be seen as limitations of the proposed CDSS.

Studies [14,15] used data from magnetic resonance spectroscopy (MRS) for automatic classification of ^1^H MR spectra from brain tumour samples. The multi-stage classifier was based on decision trees, LDA and k-NN, reduced bias and classification errors and had superior prediction capabilities [14], as compared to using only LDA in [15]. Both studies successfully categorised tumours into benign vs. malignant and low-grade vs. high-grade with higher than 90.00% classification accuracy.

Paper [16] conducted a prospective parallel-randomized pilot study to evaluate Curiam BT—a CDSS for the diagnosis of brain tumours based on ^1^H MRS. Curiam BT included four predictive models: Model 1, Model 2, Model 3 and Model 4. Model 1, with a short echo time (STE) classifier, was used to discriminate between aggressive, meningioma, and low-grade glial tumours and attained an accuracy of 88%. Model 2, with both STE and long echo time (LTE) classifiers, was used to discriminate between aggressive, meningioma, and low-grade glial tumours and achieved an accuracy of 92%. Model 3, with an STE classifier to discriminate between high-grade tumours and low-grade tumours, attained an accuracy of 83%, and Model 4 using STE to discriminate between meningiomas and non-meningioma attained an accuracy of 91%. All models were based on Fisher LDA and peak integration. The pilot study, conducted with a sample size of 55, confirmed that Curiam BT improved diagnostic accuracy and can be used as an effective tool to train and assist novice radiologists to diagnose brain tumours. To optimize the CDSS for routine practice, conducting a clinical trial with a larger sample size and integrating the CDSS within the Picture Archiving and Communication System (PACS) of the hospital are a few recommendations provided by [16].

Study [17] developed a fast spectroscopic multiple analysis (FASMA) system, based on various combinations of multiparametric MRI data for brain tumour classification. This CDSS was designed with an SVM classifier and integrated data from 3T ^1^H-MRS, DWI, DTI, and PWI for characterisation of brain tumours. The highest accuracy in classification of tumours was obtained when all the above-mentioned MR parameters were considered. It was also seen that k-NN and LDA had inferior classification accuracies as compared to the SVM classifier. SVM produced an accuracy score of >90.00% in the intra-tumoral area and >80.00% in the peri-tumoral area. FASMA provides additional information regarding tumour characteristics and can be used as an assistive tool for tumour diagnosis and grading.

Paper [18] designed a modular medical image region of interest analysis tool and repository (MIROR) for childhood cancer diagnosis. The study was conducted on a cohort of 48 children. The CDSS used advanced MRI data to differentiate between benign and malignant tumours. A 10-fold cross-validation was performed to compare the tSVM and k-NN classifiers. When utilizing all extracted features, the SVM-based classification model achieved an accuracy of 89% while k-NN-based model achieved an accuracy of 93%. The repository also aims to increase the children’s brain tumour dataset and add medical information from previous cases to assist clinicians in decision making.

The HealthAgents project, funded by the European Union, included studies [19,20,21]. The HealthAgents network is a globally distributed repository of information and knowledge regarding brain tumour diagnosis and prognosis [20]. An interactive user interface of HealthAgents was designed by [19] to facilitate classification of children’s brain tumours. The study was conducted on a cohort of 33 children with cerebellar tumours. MR spectral data was used to provide diagnostic information on brain tumours. For a three-class classifier, principal component analysis followed by LDA achieved a classification accuracy of 91.00%. A leave-one-out analysis for a two-class classifier achieved a classification accuracy of 94.00%. Through these techniques, clinicians are provided with flexibility to use MRS data for childhood brain tumour diagnosis. The first release of the HealthAgents DSS was presented in study [20]. It was based on a sample size of 182 with feature extraction performed by LDA, SVM, and LSVM. The STE and LTE models combined achieved >90.00% classification accuracy and had significant improvement over using models based on STE or LTE separately. The study concluded that in vivo MRS data, when combined with ex vivo/in vitro high-resolution magic angle spinning nuclear magnetic resonance (HR-MAS) and gene expression, has the potential to improve brain tumour classification and produce novel prognostic biomarkers [20]. A study conducted by [21] developed an independent automatic classification framework as a part of the pattern recognition technique development of the HealthAgents project. This study also suggested that including HR-MAS or gene expression data, such as DNA microarrays, could improve the diagnostic capability of the proposed framework.

The international network for pattern recognition of tumours using magnetic resonance (INTERPRET) DSS was evaluated by [22]. A multi-centre European collaboration, from 2000 to 2002 called the INTERPRET project, developed a DSS to assist neuroradiologists who had no prior experience of using MRS data to diagnose and grade brain tumours. It was seen that the tSTE classifier performed better the LTE classifier, with a classification accuracy of 89.00%. Ref. [23] evaluated the second version of the INTERPRET DSS. This study confirmed the added value of using ^1^H MRS data for brain tumour characterisation. Version 2.0 is integrated with an additional long-TE classifier as opposed to only short-TE in version 1.0. To use Version 2.0, expert knowledge was not required in spectroscopy or any specific protocol. Ref. [24] evaluated the third version of the INTERPRET DSS. It had a larger embedded database and improved diagnostic differentiation capabilities. Three LDA-based classifiers—short, long, and concatenated short+long TE— differentiated between common types of tumours. The combined LTE and STE classifier achieved the highest accuracy with 89.20%. The CDSS also successfully differentiated between tumour and pseudo-tumoral disease. The combined LTE and STE achieved a classification accuracy of 92.10%. A study by [25] evaluated the INTERPRET prototype DSS to classify brain tumours of 334 patients based on in vivo ^1^H single-voxel spectral data of different types of brain tumours. The study concluded that using MRS data for brain tumour diagnosis over MRI data alone showed significant improvement in diagnosis. The combined use of LTE and STE also improved the accuracy of classification. The LDA-based classifier integrated within this prototype DSS successfully differentiated between three tumour groups—meningiomas, LGGs and HGGs.

#### 3.3.2. Prognostic Support Systems

Study [9] was the only work identified, through this systematic review, which designed a CDSS that predicts tumour diameters under Temozolomide (TMZ) chemotherapy and provides a prognosis on when to stop treatment. The study was conducted with a sample size of 42 patients with diffuse low-grade gliomas (DLGG) and was based on two mathematical models—linear and exponential. The input variables were tumour diameters and the time of acquisition of the MRI scan since the start of the treatment. The linear model with an average accuracy of 89.00% prevailed. However, the limited number of available DLGG cases did not allow model validation on a separate dataset. Hence, increasing the size of the dataset and additionally including molecular factors that affect tumour growth are recommended.

## 4. Discussion

It should be emphasized that a CDSS does not completely substitute for a clinician’s diagnostic decision; rather, it assists clinicians in dealing with a large amount of complex medical imaging data in a shorter time span. A well-designed CDSS not only improves diagnostic capabilities but also should be easily implemented within routine clinical practice, optimizing care delivery and decision making [26].

The various types of CDSSs identified through this review for brain tumour diagnosis and prognosis were Curiam BT [16], FASMA [17], MIROR [18], HealthAgents [19,20,21] and INTERPRET [22,23,24,25]. There have been some significant achievements during the development of such systems worth mentioning. While designing and developing INTERPRET, a vast repository of brain tumours was created containing 304 histopathological STE low-grade gliomas, meningiomas, and high-grade malignant tumours. Another achievement of this project was to define a data acquisition protocol to standardize data collection from different centres. MIROR helps in providing the latest techniques and findings in the diagnosis of brain tumours improving the skillset of the clinicians.

A major area of concern is not just designing the CDSS software but also implementation and acceptance of its use by clinicians in routine clinical practice. Acceptance of CDSS use will only be possible if the clinicians view CDSS both as a tool and a process. Clinicians are also more likely to use the CDSS if their own decision-making matches with the system’s [26]. The systems’ frequent lack of transparency regarding how the output was achieved can be another reason why there is lack of user acceptance [27]. Another notable barrier to CDSS implementation is its inability to effectively support complex patient care [28]. The implementation of CDSS incurs initial costs, such as those associated with training, support, and maintenance. Overcoming the initial cost barrier is essential to realise the potential benefits of CDSS [29]. Addressing these barriers requires careful planning, stakeholder engagement, organisational readiness, and understanding of potential benefits of CDSS in improving clinical decision-making, patient outcomes, and healthcare delivery [30].

For CDSSs, as prognostic support systems, more research is needed, due to a limited number of articles in this review, to show the overall capability of such systems. In the literature, more focus has been given to designing diagnostic support systems, as opposed to prognostic support systems, which leaves the latter to be explored further.

The strength of this systematic review is that it has highlighted the importance of addressing potential drawbacks of CDSSs along with their advantages. This could lead to improved decision-making, risk mitigation, and faster implementation in clinical practice. Not bound by a timeframe to include all relevant studies and having the search strategy highly specific on CDSS used in brain tumour diagnosis and prognosis based on medical imaging data are other strengths of this review. However, a limitation should be mentioned. This review only considered studies published in the English language; there could be others CDSSs being developed that are published in different languages.

There is scope for future work that can be recommended based on this systematic review. The presence of a global standard protocol may increase CDSSs’ translation into routine clinical practice. Additionally, including clinicians’ feedback, user needs, and expectations while designing the CDSS may improve its acceptance at the point of care. Finally, if there is a stage where the CDSSs reveal their decision-making process, it will allow clinicians to increase their engagement with the systems and accelerate CDSS adoption. This type of a multi-task CDSS can be fully embedded within a clinician’s regular workflow. This will give rise to a more trainable system that can accept feedback, revise recommendations, and provide alternative clinical decisions for improved healthcare delivery.

## 5. Conclusions

Management and treatment of brain tumours require an early and accurate diagnosis, while prognostic understanding can also be beneficial in the choice of care planning for the patient. Advances in neuro-oncology imaging techniques have improved both detection and treatment planning of these tumours. Leveraging advanced imaging technologies, vastly available medical knowledge, and patient-specific information, a CDSS provides evidence-based recommendations to assist clinicians at the point of care. It reduces medical errors, enhances diagnostic capabilities, and has the potential to improve healthcare delivery. Presence of a global standard or guideline specific to CDSSs on brain tumour diagnosis and prognosis is recommended. Increased effort must be taken not only in developing such CDSSs but also when implementing them into routine clinical practice to increase clinicians’ engagement and CDSS adoption. To be able to do so, highlighting a few of areas of improvement is necessary. Although a CDSS improves diagnostic capabilities and healthcare delivery, there is a lack of specific evidence or studies to support these claims. The absence of empirical data slows down both user acceptance and evaluation of the actual impact of CDSS on brain tumour management. Embedding CDSS into routine clinical practice may increase complexity, requiring additional funding and investment in the latest technology, infrastructure, and training of healthcare professionals. With each patient condition being unique, a well-tailored patient-specific recommendation is needed, which is a drawback of current CDSSs as they lack customization. Ethical and legal considerations such as patient privacy and safety, consent, and liability should be given importance alongside the technical aspects. Instead of emphasizing the advantages of implementing CDSS, it is important to address its potential drawbacks and ethical implications. By doing so, we can promote the responsible use of CDSSs and facilitate their faster adoption in clinical settings.

## Figures and Tables

**Table 1:** Study design.

Study Design	Number of Papers	Percentage of Papers (%)
Prospective cohort study ^1^	6	35
Retrospective study	1	6
Registry-based	10	59

^1^ Comprising 1 parallel randomized pilot trial.

## Abbreviations

The following abbreviations have been used in this review.

BSVM	boosting support vector machine
CDSS	clinical decision support system
CT	computed tomography
DLGG	diffuse low-grade glioma
DSS	decision support system
DTI	diffusion tensor imaging
DWI	diffusion weighted imaging
FASMA	fast spectroscopic multiple analysis
GLCM	gray-level co-occurrence matrix
HGG	high-grade glioma
HR-MAS	high-resolution magic angle spinning nuclear magnetic resonance
INTERPRET	international network for pattern recognition of tumours using MR
k-NN	k-nearest neighbors algorithm
LDA	linear discriminant analysis
LGG	low-grade glioma
LTE	long echo time
MeSH	medical subject headings
MIROR	modular medical image region of interest analysis tool and repository
MRI	magnetic resonance imaging
MR	magnetic resonance
MRS	magnetic resonance spectroscopy
NB	naïve Bayes
NNW	neural network
PACS	picture archiving and communication system
PET	positron emission tomography
PRISMA	preferred reporting items for systematic reviews and meta-analyses
PWI	perfusion weighted imaging
QDA	quadratic discriminant analysis
STE	short echo time
SVM	support vector machine
TMZ	Temozolomide
WHO	World Health Organization
